# Epidemiological profile and main allergens identified in cases of allergic contact cheilitis^☆^

**DOI:** 10.1016/j.abd.2025.501170

**Published:** 2025-08-04

**Authors:** Bruna Cavaleiro de Macedo Souza, Laiza Bertolucci Ferrero, Mariana de Figueiredo Silva Hafner, Rosana Lazzarini

**Affiliations:** a Dermatology Clinic, Irmandade da Santa Casa de Misericórdia de São Paulo, São Paulo, SP, Brazil; b Faculty of Medical Sciences, Santa Casa de São Paulo, São Paulo, SP, Brazil

*Dear Editor,*

Allergic contact cheilitis (ACC) is an inflammatory reaction on the lips resulting from a type 4 hypersensitivity reaction.^1^ They may occur alone or in association with perioral eczema, stomatitis, contact dermatitis, atopic dermatitis, and other diseases.^1^, ^2^ The main etiologies described include: musical instruments, hygiene products (such as toothpaste and lip balm), cosmetics (applied to the face or other areas), topical medications, dental materials or food allergens.^3^

Patients from outpatient clinics of a public and a private service with suspected ACC, with exclusive involvement of the lips, treated between January 2018 and August 2024, were selected. The allergens used in the tests were those present in the Brazilian standard batteries (FDA Allergenic/RJ/Brazil), hair series and Latin American series (IPI Asac-SP/Brazil, consisting of the substances present in the standard series plus propyl gallate, disperse blue, dialkyl thiourea, methylisothiazolinone (MI), hydrocortisone butyrate, fragrance mix 2, cocamidopropyl betaine, Methyldibromo glutaronitrile (MDBGN), IPPD, sesquiterpene lactone mix, propolis, budesonide, hydrocortisone acetate, lyral, toluenesulfonamide-formaldehyde resin, Diazolidinyl urea, and imidazolidinyl urea) in addition to allergens selected from the literature that are common for allergic contact dermatitis (ACD) in this location (compounding pharmacy). The readings were performed at 48 and 96 hours, according to previously established criteria.

A total of 736 patch tests were performed in the public service and 250 in the private sector during the analyzed period, totaling 986 tests. Of these, those with lesions exclusively on the lips were selected. Thus, there were 8 cases (1%) in the public service and 13 (5.2%) in the private sector, totaling 21 cases (2.1%). The patients' ages ranged from 18 to 78 years, with a mean of 35 years. There was a higher frequency of female patients (76%). The average time from disease onset to patch testing was 12 months. The most frequent substance in the tests was Fragrance Mix 1 (FM1 14.6%), as shown in Table 1.

**Table 1:** Distribution of positive substances in patch tests in cases of Allergic Contact Dermatitis (ACD) of the lips.

Substance	Number of reactions	Percentage (%)
Fragrance mix 1	9	14.60%
Galates	5	8.20%
MDBGN	4	6.60%
Fragrance mix 2	3	4.80%
CAPB	3	4.80%
Sodium palladate	3	4.80%
Propolis	3	4.80%
Carba mix	2	3.30%
p-tertiary-butylphenol	2	3.30%
Cinnamaldehyde	2	3.30%
Lanoline	1	1.50%
Balsam of Peru	1	1.50%
Parabens	1	1.50%
Diazolidinyl urea	1	1.50%
Shellac	1	1.50%
Lyral	1	1.50%
Sesquiterpene lactone mix	1	1.50%
Eugenol	1	1.50%
Dimethicone	1	1.50%
Hydrocortisone	1	1.50%
Benzyl alcohol	1	1.50%
Chloroxylenol	1	1.50%
Formaldehyde	1	1.50%
Benzophenone 3	1	1.50%
BHT	1	1.50%
Propylene glycol	1	1.50%
MI	1	1.50%
Benzyl cinnamate	1	1.50%
Citronellol	1	1.50%
Total	62	100%

*Some patients had more than one positive test.

CAPB, Cocamidopropyl betaine; MDBGN, Methyldibromo glutaronitrile; BHT, Butylhidroxytoluiene; MI, Methylisothiazolinone.

A total of 21 suspected cases of ACC were evaluated, with lesions exclusively on the lips (2.1%) among those submitted to patch tests, showing a low frequency of the dermatosis in this location, in agreement with results in the literature, which show a frequency of 1% to 3.4%.^4^ The patients’ mean age of 35 years, ranging from 18 to 78 years, was similar to that of some studies, which showed ages ranging between 41 and 47 years. The group consisted mostly of women (16/76%), a finding consistent with other studies, possibly due to the greater use of cosmetics by this population.^4^, ^5^ The most common allergens observed were fragrances, respectively FM1 (9/14.6%) and FM2 (3/4.8%), present in toothpastes, topical medications, lip cosmetics and perfumes. Their relevance for cases of cheilitis in the literature ranged from 5.4% to 38%, consistent with the findings in this study.^6^, ^7^ Balsam of Peru, also considered a marker of fragrance allergy, was positive in one case (1.5%), which was associated with positivity to citronellal (1.5%). CAPB (Cocamidopropyl betaine), positive in three cases (4.8%; Figure 1, Figure 2), is a common lip allergen classified as a surfactant, often diluted in peppermint oil, a common ingredient in toothpastes.^8^ Gallates are antioxidants present in cosmetics, topical medications, and foods, and is also commonly used in lipsticks; they were positive in five cases (8.2%) in this study, a lower prevalence than the 36% found in the study by Loidi-Pascual et al.^4^ MDBGN is a preservative used in liquid soaps, shampoos, moisturizers, vaginal gels, makeup remover wipes, glues, adhesives, sanitary pads and topical medications; it was positive in 6.6% of cases and was related in the present patients to the use of lip balms.^5^, ^7^ Propolis is an uncommon allergen for the lips and is generally present as a contaminant in beeswax, a common component in balms and lipsticks, having been observed in one case (1.5%); however, the study by Nyman et al. showed a 19% frequency among 95 patients with suspected contact cheilitis, CD of the face and perioral region.^5^ Some preservatives, such as parabens, diazolidinyl urea and BHT (butylhidroxytoluiene), are mainly used in topical medications and are considered the second most common group of allergens in cosmetics after fragrances, with prevalence rates of up to 32%.^7^ Among the evaluated cases, they were positive in one case each (1.5%). Shellac is a natural resin from an insect (*Laccifer lacca*), used in the food, pharmaceutical and cosmetic industries for its emollient and film-forming properties and found in hair sprays, lotions, shampoos, eyeliners, mascara, nail polish, lipsticks and fragrances; it was positive in one case (1.5%) and was related to lipstick use.^3^ Finally, benzophenone-3 is a component of sunscreens, also present in other cosmetics with the function of protecting the product against deterioration due to ultraviolet radiation action; among the study patients, it was positive in one case (1.5%), related to the use of lip gloss.

**Figure 1:**
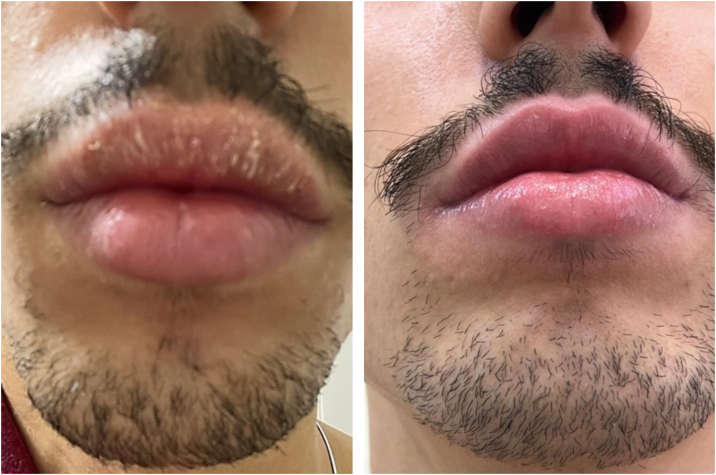
Patient with Allergic Contact Cheilitis (ACC) on the lip with sensitivity to CAPB (Cocamidopropyl betaine). Left: patient at the first consultation. Right: patient after stopping contact with CAPB.

**Figure 2:**
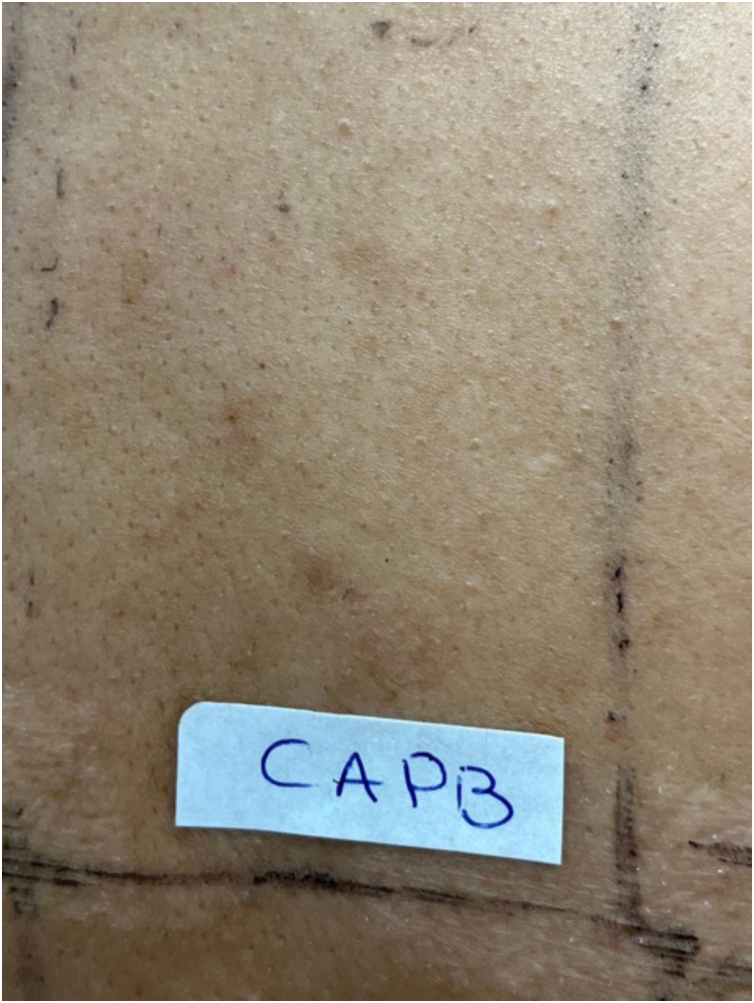
Patch test on the back with positivity to CAPB (Cocamidopropyl betaine) for the patient with Allergic Contact Cheilitis (ACC) on the lip.

Even though the literature describes metals (e.g., nickel sulfate and potassium bichromate) and drugs (e.g., neomycin) as allergens frequently implicated in contact cheilitis, these findings were not evidenced in the present study, probably because of the small sample size.^9^

Although the patch tests in this study were not performed with the products used by the patients, as recommended in some studies, a detailed anamnesis was performed to ensure that the allergens to which the patients were exposed were included in the battery of applied tests.^9^ Although patients are instructed to bring the suspected products on the day of the test, there is low adherence to the procedure, often with claims of forgetting or not having the product at the time of the exam.

Although the group studied a small number of patients, the study demonstrates the importance of patch tests in cases of cheilitis, to establish the diagnosis of allergic contact dermatitis, as well as the most prevalent causative agents.^9^

## Financial support

None declared.

## Authors’ contributions

Bruna Cavaleiro de Macedo Souza: Statistical analysis; approval of the final version of the manuscript; design and planning of the study; drafting and editing of the manuscript; collection, analysis and interpretation of data; effective participation in research orientation; intellectual participation in the propaedeutic and/or therapeutic conduct of the studied cases; critical review of the literature; critical review of the manuscript.

Laiza Bertolucci Ferrero: Statistical analysis; approval of the final version of the manuscript; drafting and editing of the manuscript; collection, analysis and interpretation of data; critical review of the literature; critical review of the manuscript.

Mariana de Figueiredo Silva Hafner: Statistical analysis; approval of the final version of the manuscript; design and planning of the study; drafting and editing of the manuscript; collection, analysis and interpretation of data; effective participation in research orientation; intellectual participation in the propaedeutic and/or therapeutic conduct of the studied cases; critical review of the literature; critical review of the manuscript.

Rosana Lazzarini: Statistical analysis; approval of the final version of the manuscript; design and planning of the study; drafting and editing of the manuscript; collection, analysis and interpretation of data; effective participation in research orientation; intellectual participation in the propaedeutic and/or therapeutic conduct of the studied cases; critical review of the literature; critical review of the manuscript.

## Conflicts of interest

None declared.
